# What Moves Them? Active Transport among Inhabitants of Dutch Deprived Districts

**DOI:** 10.1155/2013/153973

**Published:** 2013-09-26

**Authors:** Carla Saris, Stef Kremers, Patricia Van Assema, Cees Hoefnagels, Mariël Droomers, Nanne De Vries

**Affiliations:** ^1^Caphri School of Public Health and Primary Care, Maastricht University, P.O. Box 616, 6200 MD Maastricht, The Netherlands; ^2^Faculty of Health Medicine and Life Sciences, Department of Health Promotion, Maastricht University, P.O. Box 616, 6200 MD Maastricht, The Netherlands; ^3^NUTRIM, School for Nutrition, Toxicology and Metabolism, Maastricht University, P.O. Box 616, 6200 MD Maastricht, The Netherlands; ^4^Trimbos Institute, Netherlands Institute for Mental Health and Addiction, P.O. Box 725, 3500 AS Utrecht, The Netherlands; ^5^Faculty of Medicine, Department of Public Health, University of Amsterdam, P.O. Box 19268, 1000 GG Amsterdam, The Netherlands

## Abstract

*Background*. Active modes of transport like walking and cycling have been shown to be valuable contributions to daily physical activity. The current study investigates associations between personal and neighbourhood environmental characteristics and active transport among inhabitants of Dutch deprived districts. *Method*. Questionnaires about health, neighbourhoods, and physical activity behaviour were completed by 742 adults. Data was analysed by means of multivariate linear regression analyses. *Results*. Being younger, female, and migrant and having a normal weight were associated with more walking for active transport. Being younger, male, and native Dutch and having a normal weight were associated with more cycling for active transport. Neighbourhood characteristics were generally not correlated with active transport. Stratified analyses, based on significant person-environment interactions, showed that migrants and women walked more when cars did not exceed maximum speed in nearby streets and that younger people walked more when speed of traffic in nearby streets was perceived as low. Among migrants, more cycling was associated with the perceived attractiveness of the neighbourhood surroundings. *Discussion and Conclusion*. Results indicated that among inhabitants of Dutch deprived districts, personal characteristics were associated with active transport, whereas neighbourhood environmental characteristics were generally not associated with active transport. Nevertheless, interaction effects showed differences among subgroups that should be considered in intervention development.

## 1. Introduction

Overweight and obesity prevalence rates are increasing worldwide. About 1.4 billon adults were overweight in 2008, of whom 200 million men and 300 million women were considered to be obese [1]. In The Netherlands, prevalence rates of overweight (and obesity) are 60% (14%) in men and 44% (13%) in women [2]. High prevalence rates have been found specifically among ethnic minorities and low socioeconomic status (SES) groups [1, 3]. 

Regular physical activity can attenuate health risks associated with overweight [4]. These health risks include cancer, cardiovascular diseases like hypertension and diabetes, and problems with mobility [5–7]. The WHO [8] recommends adults aged 18–64 to engage in moderate-intensity aerobic physical activities such as walking, cycling, or doing household chores for at least 150 minutes a week. Active transport (i.e., walking or cycling from A to B) can thus help achieve sufficient levels of physical activity [9, 10]. This study therefore focuses on the factors associated with active transport.

Bauman et al. [11] investigated demographic factors as correlates of physical activity. This review of reviews showed that age and overweight were inversely correlated with physical activity, whereas the male gender and ethnic origin (white) were positively correlated with being active. Generally, these correlates were also found for active transport among low-income populations [12]. In the past decade, research has also focused on environmental correlates of physical activity. Particularly, studies from the USA and Australia have found positive relations between physical activity and neighbourhood walkability [13, 14]. Most of these studies, however, have been performed among high-SES populations who tend to live in neighbourhoods with relatively high walkability. Moreover, high-SES populations have shown higher levels of physical activity and lower prevalence rates of overweight compared to low-SES populations. It is not yet clear whether the findings from these studies are also valid for low-SES populations living in deprived neighbourhoods [12]. Detailed information on the relation between neighbourhood characteristics and physical activity among subgroups within this population is currently lacking. It is hypothesized that more favourable neighbourhood characteristics are associated with higher levels of activity.

Past studies that focused on the effect of neighbourhood walkability on active transport have shown that associations are reduced after controlling individual demographic characteristics [15]. However, in addition to the confounding role of demographic characteristics, knowledge about interactions between demographic variables and neighbourhood characteristics is required in order to distinguish subgroups that are more vulnerable or responsive to environmental features than others. This could help to define specific target groups for environmental interventions. 

Inhabitants of Dutch deprived neighbourhoods vary in ethnic background and are often characterized by a low socioeconomic status [16]. As a result, the prevalence rate of overweight is higher, and level of physical activity is lower compared to those of the average Dutch population [17, 18]. This makes inhabitants of Dutch deprived districts an important target group for physical activity interventions. Furthermore, previous research has shown that it is difficult to address ethnic minorities, and guidelines for this specific group are lacking [19]. More insight into the personal and neighbourhood factors that are associated with active transport could contribute to the development of effective interventions targeting ethnic minorities. Therefore, this study investigates which personal and neighbourhood environmental characteristics are associated with active transport among inhabitants of Dutch deprived neighbourhoods. In addition, interactions between personal and neighbourhood environmental characteristics will be tested to explore differential associations of neighbourhood walkability with active transport within subgroups.

## 2. Method

### 2.1. Data Collection

Data are derived from the URBAN40 study. Briefly, URBAN40 is a longitudinal study that evaluates the health impact of area-based interventions to improve housing, employment, education, social integration, and safety in the 40 most severely deprived neighbourhoods in The Netherlands, also known as the Dutch District Approach [16]. In 2007, the Dutch government made a list of the 140 most deprived neighbourhoods in the Netherlands, based on scores on eighteen different registry-based indicators of physical and socioeconomic deprivation as well as physical and social problems reported by residents [20]. From this list, twenty neighbourhoods were selected that belonged to the 140 most deprived Dutch neighbourhoods. In each district, 250 randomly selected adults aged 18 and older received a letter in which they were invited to participate, including a reply card that they could return free of charge. Trained interviewers contacted the residents who sent a reply card and arranged home visits for the interview. Up to two reminder letters were sent when people did not respond via the reply card. In addition, trained interviewers from an interview agency specialized in research among multicultural populations recruited participants by unplanned home visits (ringing doorbell at the addresses on the original list). The trained interviewers were tailored to gender and ethnicity (spoken language, e.g., Dutch, English, Turkish, Classical Arabic, or Arab-Berber) of the residents. Financial constraints limited recruitment of respondents by unplanned home visits in all districts, favouring larger districts. 

Respondents filled out the questionnaire by themselves, but they could also ask the assistant that brought the questionnaire to help them, if needed in their own language (Dutch, English, Turkish, Classical Arabic, or Arab-Berber). In particular the participants that were of non-Dutch origin made use of this option; they were orally interviewed and the assistant filled in the answers. 

### 2.2. Study Sample

A total of 5000 respondents were invited to participate. Of the 374 participants that returned the reply card, 299 filled out the questionnaire. Another 441 participants were recruited by means of home visits. This resulted in a total study population of 740 adults that were recruited between May 2010 and November 2011. After deletion of cases without data on key variables (*n* = 118), a final sample of 622 participants remained. 

### 2.3. Measures

Active transport was defined as the minutes per week spent on walking or cycling from A to B. The validated SQUASH questionnaire for physical activity measured active transport by asking respondents to think about a regular week in the past months [21]. Respondents then indicated how many days per week they engaged in several forms of physical activity and how many minutes they engaged in them. Subsequently, they could fill in the days, hours, and minutes spent on active transport. Total minutes of walking for active transport per day were calculated by multiplying the number of hours reported with sixty and adding them to the minutes reported. Subsequently, minutes of active walking per week were calculated by multiplying the minutes per day with the reported number of days per week. This number of minutes of walking for active transport per week was used in the analyses. Minutes of cycling for active transport per week were calculated according to the same steps.

Neighbourhood environmental characteristics were assessed by use of the Neighbourhood Walkability Scale (NEWS) [22]. Three scales with twelve questions in total investigated the access to services (three items) (*α* = 0.71) (e.g.,* shops are within easy walking distance from my home)*, neighbourhood surroundings (four items) (*α* = 0.66) (e.g., *the natural surroundings in my neighbourhood are beautiful), *and safety from crime (five items) (*α* = 0.66) (e.g., *because of criminality it is unsafe to walk in my neighbourhood during the day)*. Three items of the “safety from traffic” scale were included separately in the analyses because of the low reliability of the scale (*α* < 0.6). The items were “there is much traffic on nearby streets which make walking in the neighbourhood difficult or unpleasant;” “the speed of traffic in nearby streets is normally low (30 km/h or less);” and “most car drivers exceed maximum speed when driving through the neighbourhood.” The answers were given on a four-point Likert scale (“completely disagree” (1) to “totally agree” (4)). A higher score corresponded with better access to services, better neighbourhood surroundings, and more safety from crime and traffic. 

Personal characteristics that were taken into account were age, sex, ethnicity, and body mass index (BMI). Ethnicity of the respondent was operationalized by assessing the country of birth of both parents. If a respondent had at least one parent who was born outside The Netherlands, he or she was considered to be a migrant [23]. BMI was calculated by self-reported measures of height and weight [24]. Neighbourhood status was included as a confounder, to correct variations in degree of deprivation among neighbourhoods. Neighbourhood status was determined using the Neighbourhood Status Score (NSS) of the Dutch Social Cultural Planning Office (SCP). This score is based on the income, education level, and employment status of the inhabitants and indicates the degree of social deprivation per neighbourhood. Scores range between −4 and +4 [25]. Lower scores correspond with a larger degree of social deprivation, whereas high scores indicate a more favourable socioeconomic status. 

### 2.4. Statistical Analyses

Characteristics of the target population were investigated by means of descriptive analyses. Independent samples *t*-tests were performed to investigate the difference in scores on the NEWS and in minutes of active transport per week between the following subgroups: respondents equal to or younger than the median age of 43 years and respondents older than 43 years: men and women; native Dutch respondents and migrants; and respondents with a BMI lower than 25 and respondents with a BMI higher than 25. 

Bivariate correlations were calculated to explore the associations between personal characteristics, and minutes per week spent in active transport and the associations between NEWS scales and minutes spent engaging in active transport. In order to gain more insight into these correlations and to adjust for the difference in effect between baseline characteristics multivariate linear regression analyses were performed. The full main effects model consisted of NSS, the personal characteristics (age, sex, ethnicity, and BMI), and the neighbourhood characteristics. Interactions between personal characteristics and neighbourhood variables were investigated by adding each interaction term separately to the full main effects model. Stratified analyses were performed to explore subgroup effects in case of significant interaction terms. 

## 3. Results

### 3.1. Descriptives

The mean age of the respondents was approximately 44 years (Table 1). Slightly more than half of them were women. Most of the participants were Dutch, the migrants were mainly non-Western (87.45%; not tabulated). More than half of the study population (52.9%) was overweight or obese. The NSS of the twenty participating districts varied between −3.64 (highly deprived) and −0.15 (moderately deprived) with a mean of −1.82 and a SD of 1.03.

Table 2 shows the scores for the neighbourhood characteristics and the minutes of active transport per week for subgroups of the population. On average, residents of deprived neighbourhoods walked 33 minutes and cycled for 32 minutes per week for active transport. The younger participants were found to be more active in terms of walking as well as cycling than the older participants. Migrants were more active in terms of walking, whereas the Dutch biked more often. Participants with a BMI below 25 were more active than those overweight, with a significant difference in activity levels for cycling. 

Respondents older than 43 years perceived the neighbourhood surroundings as more positive than the younger respondents. Women experienced better access to services than men. Native Dutch participants perceived better access to services, better neighbourhood surroundings, and felt safer from crime than migrants. Furthermore, they experienced less traffic on nearby streets that made walking difficult than migrants. Participants who lived in a neighbourhood with a relatively higher status within this population perceived all neighbourhood characteristics as more favourable than the people from districts with a lower NSS. 

### 3.2. Walking for Active Transport

Multivariate regression analyses showed that minutes of walking decreased significantly by one minute for each year increase in age. NSS was also found to be a significant associate of walking; respondents from higher status neighbourhoods walked less than respondents from lower status neighbourhoods. Perceived speed of traffic was the only neighbourhood variable found to be a statistically significant associate of walking. Respondents walked more when the perceived speed of traffic was low.

Interactions between ethnicity and the perception of traffic exceeding maximum speed (*β*: 0.40; *P* < 0.001); sex and the perception of traffic exceeding maximum speed (*β*: 0.26; *P* < 0.05); and between age and speed of traffic (*β*: −0.53; *P* < 0.01) were statistically significant. Subgroup analyses indicated that migrants and women reported more walking for transport when cars were perceived to not exceed maximum speed (*β*: 0.18; *P* < 0.01 and *β*: 0.13; *P* < 0.05, resp.), whereas this perceived environmental factor was unrelated to walking of native Dutch and male respondents (*β*: −0.06; ns and *β*: −0.04; ns, resp.). Stratification for age showed that low perceived speed of traffic was significantly associated with more walking in respondents of 43 years or younger (*β*: 0.18; *P* < 0.01) but not in respondents older than 43 (*β*: −0.06; ns). 

### 3.3. Cycling for Active Transport

Every year of increase in age was associated with a decrease of one minute of cycling per week (Table 3). The native Dutch cycled more compared with the migrants (*β*: −0.11; *P* ≤ 0.01). Neighbourhood walkability was not related to cycling for active transport. 

The interaction between ethnicity and neighbourhood surroundings was found to be significant (*β*: 0.50; *P* ≤ 0.01). More attractive neighbourhood surroundings were significantly associated with more cycling per week among the migrants (*β*: 0.23; *P* ≤ 0.001), but not in the Dutch respondents (*β*: −0.02; ns). 

## 4. Discussion

This study investigated which personal and neighbourhood environmental characteristics were associated with active transport among inhabitants of Dutch deprived neighbourhoods. Younger people spent more time walking and cycling for transport. This finding is supported by a study of Ogilvie et al. [12] and could be a result of differences in fitness between the younger and the older respondents. People tend to perceive more barriers for physical activity as they age, such as physical disabilities and poorer perceived health [26]. Migrants walked more minutes per week compared with the Dutch, probably because walking is considered to be an easy and cheap mode of transportation, which is less sensitive to cultural habits compared to cycling [27]. The Dutch cycled more than the migrants, probably because they are more used to cycling and have more access to this mode of transportation [28]. Higher scores on BMI correlated with lower levels of active transport, which confirms findings of Pucher et al. [29]. 

Perceived speed of traffic was the only neighbourhood environmental characteristic that was associated with walking for transport. When the average speed of traffic was perceived as low, people reported more walking for active transport. A review of previous studies reported mixed evidence with regard to safety and active transport, which illustrates a need for clear definitions of safety in this field of research [30]. Our measurements confirm this need for proper measurements of safety from traffic, since we were forced to include the relevant questions separately in the analyses as a result of low reliability of the scale.

Interactions showed that with regard to ethnicity, migrants appeared to be more responsive to neighbourhood walkability than the Dutch respondents. Speed of traffic (walking) and aesthetics (cycling) was found to be more strongly associated with active transport among migrants than among native Dutch. This could be a result of cultural differences; migrants seem to be more in need of a safe, attractive, and stimulating environment to induce active transport [31]. An investigation of transportation among Dutch migrants showed that migrants cycled more when high quality cycle infrastructure was available and if a strong cycle culture was present in the neighbourhood [31]. In addition, female and younger respondents were subgroups that appeared to be somewhat more responsive to traffic safety than male and older respondents. A Dutch study on traffic safety found that higher levels of neighbourhood traffic safety correlated with increased odds of being active, especially in women and people aged 35 to 59 [32], which partly confirms our findings. 

Personal characteristics have been shown to be stronger associates of active transport than neighbourhood characteristics, a finding which is supported by multiple other studies [33, 34]. Nevertheless, there were also studies that did find associations between neighbourhood walkability and active transport [13, 14]. This could be explained by differences in levels of transportation facilities in the countries under study. Most of these studies were conducted in the US or Australia, countries that sometimes lack proper walking or cycling trails. However, The Netherlands especially is already well-equipped for facilitation of walking and cycling, so relative differences in neighbourhood characteristics may only have a minimal impact on active transport. This implies that results of these studies are likely to be only valid for the population and country in which it was conducted, as was suggested in previous studies [12, 34]. The low variability of neighbourhood characteristics within the deprived districts in our study may have decreased the possibility to find strong environmental relationships even more. It is also possible that the characteristics investigated in this study did not cover all environmental aspects that might be relevant in active transport. Nevertheless, interaction effects indicated that within some groups of the population under study, neighbourhood characteristics are important and need to be considered in efforts to promote physical activity. 

Some limitations need to be considered when interpreting the results. Since the data collected for this study was cross-sectional, statements about causality cannot be made. Second, the overall response rate was low, although it was equivalent to those of comparable studies of the past years [10, 12, 35]. Third, self-reported measures of active transport were used, which could have been prone to recall bias and socially desirable answers. Fourth, an abbreviated version of the NEWS questionnaire was used, which may be the reason for the one scale on safety from traffic being considered unreliable. 

Despite some limitations, this study adds valuable information to current evidence in particular because of the focus on a low-SES population, on the understudied cycling behaviour [33], and the ethnic composition of the study population. Participants were hard to reach and are therefore often neglected in research, among others because of possible cultural and language differences between the interviewer and respondents. Intensive recruitment strategies such as matching of interviewer and participant and executing the study in the respondents' mother tongue were used to overcome this difficulty. The setting of deprived neighbourhoods was also a valuable characteristic, since they are the focus of governmental policies and interventions. Insight into the associations between neighbourhood characteristics and patterns of physical activity and their determinants in these districts could provide more information about the contents and implementation of such policies and interventions. 

## 5. Conclusion

Active transport among inhabitants of Dutch deprived districts is mostly associated with personal characteristics. However, neighbourhood characteristics were found to be important in certain subgroups as well. Results of the current study may be used to design experimental research in order to test causality of the findings. Eventually, this may lead to evidence for effective intervention development for the subgroups most in need of interventions to increase active transport and subsequently levels of physical activity.

## Figures and Tables

**Table 1:** Population characteristics.

Demographics	Total sample (*N* = 622)
Neighbourhood status score range (median)	−3.64 to −0.15 (−2.03)
Mean age in years (SD)	43.97 (14.57)
Gender	
Men (%)	285 (45.8%)
Women (%)	337 (54.2%)
Ethnicity	
Dutch (%)	375 (60.3%)
Migrant (%)	247 (39.7%)
BMI (weight/length^2^)	
<18.5 (%)	9 (1.4%)
≥18.5 to <25 (%)	284 (45.7%)
≥25 to <30 (%)	225 (36.2%)
≥30 (%)	104 (16.7%)

**Table 2:** Neighbourhood characteristics and minutes of active transport for subgroups.

	Mean scores neighbourhood variables (SD)						Mean minutes per week (SD)	
	Access to services (0–12)	Neighbourhood surroundings (0–16)	Safety from crime (0–20)	Many traffic (0–4)	Speed of traffic (0–4)	Traffic exceeds maximum speed (0–4)	Walking (active transport)	Cycling (active transport)
Total	10.55 (1.68)	10.86 (2.60)	15.94 (2.75)	2.84 (0.85)	2.63 (0.93)	2.26 (0.88)	33.01 (73.33)	32.37 (67.88)
Age ≤ 43 (*n* = 315)	10.50 (1.70)	10.66 (2.66)	15.99 (2.62)	2.84 (.83)	2.27 (.87)	2.64 (.91)	44.41 (82.10)	41.44 (74.46)
Age > 43 (*n* = 307)	10.62 (1.67)	11.07 (2.52)*	15.88 (2.89)	2.84 (.86)	2.26 (.89)	2.62 (.96)	21.31 (61.02)***	23.07 (59.08)***
Men (*n* = 285)	10.38 (1.84)	10.73 (2.57)	16.00 (2.74)	2.81 (.85)	2.66 (.93)	2.22 (.89)	31.48 (73.27)	35.54 (72.87)
Women (*n* = 337)	10.70 (1.52)*	10.97 (2.62)	15.89 (2.77)	2.87 (.84)	2.61 (.94)	2.30 (.87)	34.30 (73.46)	29.69 (63.34)
Ethnicity Dutch (*n* = 375)	10.69 (1.66)	11.18 (2.55)	16.29 (2.72)	2.90 (.83)	2.58 (.98)	2.23 (.88)	25.81 (63.46)	37.80 (70.47)
Ethnicity migrant (*n* = 247)	10.35 (1.70)*	10.37 (2.60)***	15.41 (2.72)***	2.74 (.85)*	2.70 (.85)	2.32 (.87)	43.93 (85.16)**	24.14 (62.99)*
BMI ≤ 25 (*n* = 293)	10.58 (1.66)	10.83 (2.59)	15.94 (2.76)	2.91 (.82)	2.56 (.94)	2.29 (.85)	38.59 (77.38)	39.61 (71.00)
BMI > 25 (*n* = 329)	10.53 (1.71)	10.88 (2.61)	15.94 (2.75)	2.78 (.86)*	2.69 (.92)	2.24 (.91)	28.03 (69.25)	25.93 (64.40)*

*P ≤ .05, **P ≤ .01, ***P ≤ .001.

**Table 3:** Multivariate regression regarding minutes per week of walking and cycling for active transport.

Walking (active transport)	Walking active transport			Cycling active transport		
	*B *	SE *B *	*β*	*B *	SE *B *	*β*
Constant	47.00	31.39		75.42	28.98	
NSS	−17.57	5.95	−.12**	9.11	5.50	.07
Age	−1.00	0.21	−.20***	−.95	0.20	−.20***
Sex	3.37	5.80	.02	−3.83	5.35	−.03
Ethnicity	9.43	6.18	.06	−14.80	5.70	−.11**
BMI	0.11	0.68	.01	−1.05	0.63	−.07
Access to services	0.24	1.80	.01	0.46	1.66	.01
Neighbourhood surroundings	−.98	1.22	−.04	1.63	1.13	.06
Safety from crime	.74	1.15	.03	−0.12	1.06	−.01
Many traffic	1.28	3.66	.02	1.81	3.38	.02
Speed of traffic	6.34	3.23	.08*	4.27	2.98	.06
Exceed maximum speed	2.87	3.43	.03	−3.45	3.16	−.05

*P ≤ .05, **P ≤ .01, ***P ≤ .001.
